# Effectiveness of chemical pleurodesis in spontaneous pneumothorax recurrence prevention: a systematic review

**DOI:** 10.1136/thoraxjnl-2015-207967

**Published:** 2016-11-01

**Authors:** R J Hallifax, A Yousuf, H E Jones, J P Corcoran, I Psallidas, N M Rahman

**Affiliations:** 1 Oxford Centre for Respiratory Medicine, Oxford University Hospitals NHS Trust, Oxford, UK; 2 Faculty of Health Sciences, School of Social and Community Medicine, University of Bristol, Bristol, UK

**Keywords:** Pleural Disease, Thoracic Surgery

## Abstract

**Objectives:**

Spontaneous pneumothorax is a common pathology. International guidelines suggest pleurodesis for non-resolving air leak or recurrence prevention at second occurrence. This study comprehensively reviews the existing literature regarding chemical pleurodesis efficacy.

**Design:**

We systematically reviewed the literature to identify relevant randomised controlled trials (RCTs), case–control studies and case series. We described the findings of these studies and tabulated relative recurrence rates or ORs (in studies with control groups). Meta-analysis was not performed due to substantial clinical heterogeneity.

**Results:**

Of 560 abstracts identified by our search strategy, 50 were included in our systematic review following screening. Recurrence rates in patients with chest tube drainage only were between 26.1% and 50.1%. Thoracoscopic talc poudrage (four studies (n=249)) provided recurrence rates of between 2.5% and 10.2% with the only RCT suggesting an OR of 0.10 compared with drainage alone. In comparison, talc administration during video-assisted thoracic surgery (VATS) from eight studies (n=2324) recurrence was between 0.0% and 3.2%, but the RCT did not demonstrate a significant difference compared with bleb/bullectomy alone. Minocycline appears similarly effective post-VATS (recurrence rates 0.0–2.9%). Prolonged air leak and recurrence prevention using tetracycline via chest drain (n=726) is likely to provide recurrence rates between 13.0% and 33.3% and autologous blood patch pleurodesis (n=270) between 15.6% and 18.2%.

**Conclusions:**

Chemical pleurodesis postsurgical treatment or via thoracoscopy appears to be most effective. Evidence for definitive success rates of each agent is limited by the small number of randomised trials or other comparative studies.

**Key messages**

**What is the key question?**

- How effective are chemical pleurodesis agents at recurrence prevention in spontaneous pneumothorax?

**What is the bottom line?**

- Talc poudrage at thoracoscopy and talc or minocycline pleurodesis as an adjunct to surgery provide low recurrence rates. Less invasive options include pleurodesis using tetracycline or ‘blood patch’ via chest drain.

**Why read on?**

- This review is the first to systematically assess the evidence for pleurodesis efficacy in recurrence prevention for all chemical pleurodesis agents in cases of spontaneous pneumothorax in both ‘medical’ pleurodesis (ie, no intervention on the lung) and as an adjunct to surgical procedures.

## Introduction

Pneumothorax, air in the pleural space, is a common pathology. Primary spontaneous pneumothorax (PSP) refers to patients with no underlying lung disease, while those with established lung pathology are classified as secondary spontaneous pneumothorax (SSP). The incidence of spontaneous pneumothoraces based on populations in the USA^1^ and Sweden^2^ is reported as 18–24 per 100 000 cases per annum for men and 1.2–6 per 100 000 for women. PSP has a reported incidence of 7.4–18 cases (age-adjusted incidence) and 1.2–6 cases per 100 000 population per year for males and females, respectively.^1^ ^2^ UK data on hospital admission rates (for PSP and SSP combined) demonstrate an incidence of 16.7 cases per 100 000 for men and 5.8 cases per 100 000 for women, with corresponding mortality rates of 1.26 per million and 0.62 per million per annum.^3^ More recent data from France have demonstrated a similar rate of 22.7 cases per 100 000 population.^4^

Recurrence rates for spontaneous pneumothorax (SP) are quoted as approximately 30%, with individual studies reporting a recurrence rate of between 17% and 49%.^5–12^ Initial treatment regimens and strategies for recurrence prevention remain controversial. Recurrence prevention involves an attempt at pleurodesis (permanent apposition of the visceral and parietal pleura to seal the pleural space), which can be chemical (or ‘medical’) using an agent introduced into the pleural cavity or surgical by apical pleurectomy or pleural abrasion. International guidelines currently recommend pleurodesis for non-resolving pneumothoraces acutely or electively to prevent recurrence after a second occurrence of pneumothorax. However, guidelines do not specify the optimal pleurodesis approach or agent for chemical pleurodesis.^13–15^

For PSP, the American College of Chest Physicians (ACCP) Delphi consensus statement^13^ recommends surgical pleurodesis via thoracoscopy (including bullectomy) for ongoing air leak (>4 days) or recurrence prevention at second occurrence. In this statement, there was no consensus on the utility of additional talc poudrage at surgical procedure for patients with PSP. Chemical pleurodesis via a chest drain was thought to be acceptable for patients in whom surgery was contraindicated or patients who refused an operative procedure. The statement recommends doxycycline or talc as the preferred agent in cases where chemical pleurodesis is conducted.^13^ For SSP, this statement suggests intervention to prevent pneumothorax recurrence at first occurrence (in contrast to PSP), with the surgical approach as first choice and chemical pleurodesis as an option for the high-risk patient or those declining surgery. The ACCP guidance was published in 2001, and therefore may no longer be up to date.

The British Thoracic Society (BTS, 2010)^14^ and the Belgian Society of Pulmonology (BSP, 2005)^15^ guidelines for PSP and SSP both recommend surgical pleurodesis for ongoing air leak acutely and recurrence prevention at second occurrence. They state that “with the advent of VATS for pneumothorax repair and recurrence prevention, the use of surgical chemical pleurodesis has declined significantly”.^14^ Medical chemical pleurodesis is recommended in patients unwilling or unable to undergo surgery, and is therefore more likely to be applicable to patients with SSP. The BTS makes reference to tetracycline as the previous first-line agent for PSP and SSP, but with decline in usage through difficulties in supply in favour of graded talc, with passing mention of minocycline and doxycycline efficacy in animal models.^14^ The BSP does not comment on pleurodesis agent.

This study aimed to systematically review the existing literature regarding the efficacy of chemical pleurodesis for recurrence prevention in pneumothorax.

## Methods

### Eligibility criteria

We systematically reviewed the literature to identify relevant randomised controlled trials (RCTs), case–control studies and case series (without comparator groups) of ≥10 cases. Case series were specifically included because the authors were aware of a dearth of trials data in this area.

Studies were considered eligible for inclusion with the following criteria: adult patients (≥18 years old) with spontaneous (primary and secondary) pneumothorax, undergoing pleurodesis at first occurrence or subsequent recurrence, or for the treatment of persistent air leak, by instillation via chest tube or in addition to surgical procedure; interventions consisting of chemical pleurodesis with any agent. Comparators included were any of chest tube drainage alone (no pleurodesis), other pleurodesis agent and surgical procedures (eg, mechanical abrasion, bleb/bullectomy, pleurectomy). The outcome was pneumothorax recurrence rate (ideally, after at least 1 year of follow-up).

Exclusions consisted of the following: animal or paediatric studies, non-primary studies (ie, letters, editorials and review articles), pleurodesis for malignant pleural effusion, surgical pleurodesis only (with no sclerosant inserted), pleurodesis for postoperative air leak, insufficient data on agent or technique used, inadequate follow-up period (ie, <3 months) and case series with <10 cases.

Papers were also excluded if the authors were unable to obtain a translation (if not published in English) or unable to obtain the paper online or via our hosts' extensive library access collections.

### Search strategy

Literature searches of multiple databases (including PubMed, Embase, Medline, Web of Science, Cochrane Library) were performed up to and including June 2016. Results were not restricted by year of publication. Combinations of search terms were used and adapted for each database as appropriate, including “pleurodesis”, “spontaneous”, “pneumothor*”, “chemical”, “talc”, “tetracycline”, “minocycline”, “iodopovidine” and “blood”. In addition to electronic database searches, reference lists, relevant textbooks and review articles were hand-searched and back-referenced (ie, reference lists of review articles examined for additional studies not appearing in initial searches). Abstracts were independently reviewed for relevance by two authors (RJH and AY). Any discrepancies were resolved by discussion (with JPC and IP) with a low threshold for review of the full article. Relevant full journal articles were subsequently assessed again for eligibility.

### Data extraction

Data were extracted from the full articles separately by two authors using a prespecified extraction form (Microsoft Excel 2010, Microsoft, USA). Extracted information included lead author, year, geographical area, nature of pneumothorax (primary or secondary, where available), number of participants, intervention agent(s), control/comparator measures, recurrence rate for each arm, follow-up timescale (mean or median when described), study type and quality. In those with mixed populations (eg, including patients with pleural effusions or postoperative air leak), only data pertaining to SP were extracted. Where available, data on number of episodes of pneumothorax (rather than number of patients) were extracted. Early pleurodesis failures that required further procedure or surgical referral were included in the calculated recurrences rates.

### Quality and risk of bias assessment

Risk of bias of the included RCTs was assessed using the Cochrane Risk of Bias tool.^16^ This tool addresses seven domains: sequence generation, allocation concealment, blinding of participants and personnel, blinding of outcome assessment, incomplete outcome data, selective outcome reporting and ‘other issues’. We did not formally assess risk of bias in case–control studies or case series.

### Data analysis

Given the considerable heterogeneity in study design, pleurodesis agents, control groups and outcomes across identified studies, formal data synthesis via meta-analysis was not conducted as any results would not be clinically meaningful. ORs with 95% CIs were calculated where possible as a measure of the effectiveness in reducing pneumothorax recurrence relative to a control. For studies with no comparison group, we display simply estimates of the pneumothorax recurrence rate in the single arm, with a 95% CI (calculated on the log scale). When zero recurrences occurred, we approximated the upper limit of the CI for recurrence rate by 3/n^16^ (Section 16.9.4) and applied the standard approximation of adding 0.5 to all cell counts before calculating ORs and their CIs^16^ (Section 16.9.2).

## Results

**Figure 1:**
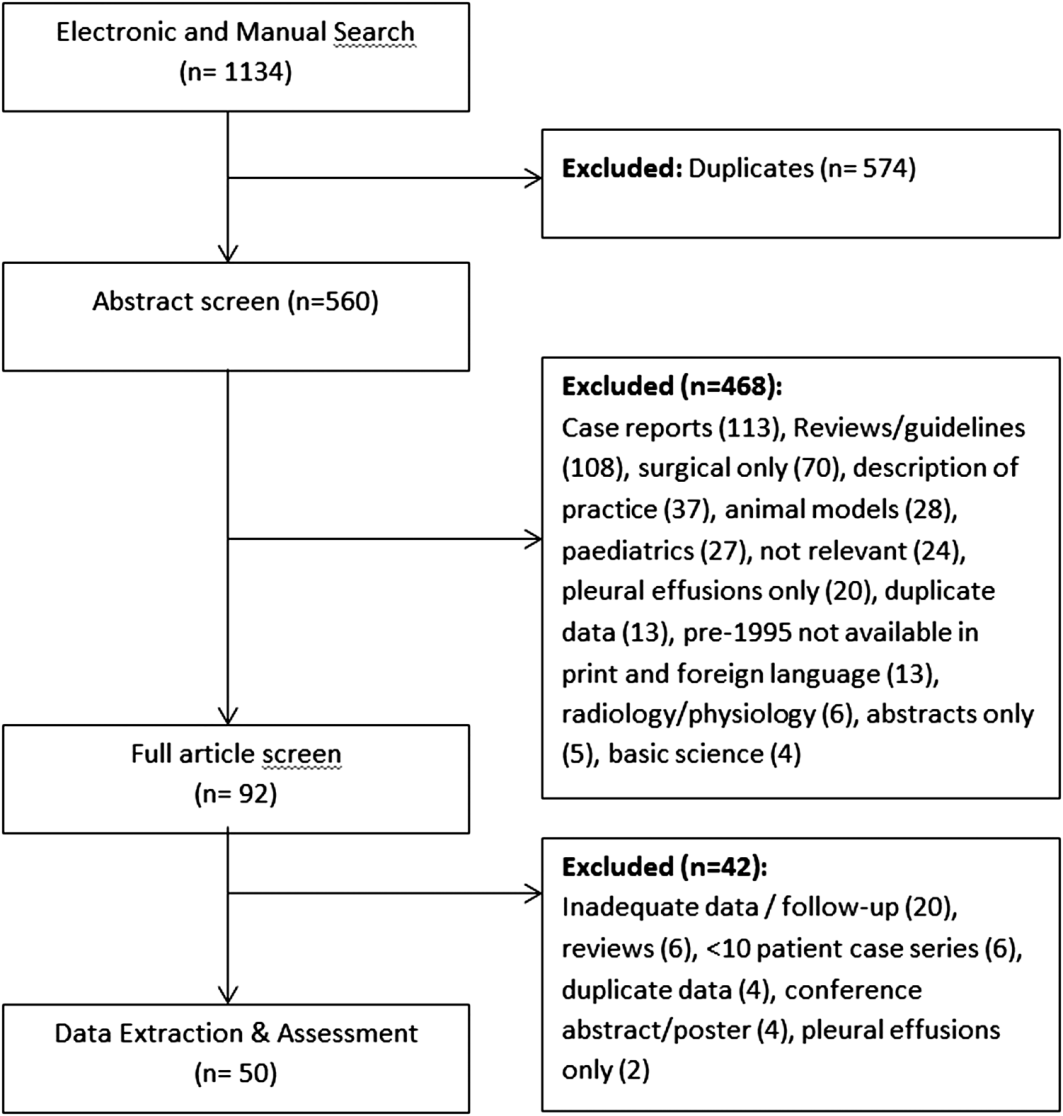
Preferred reporting items for systematic reviews and meta-analyses (PRISMA) flow diagram of study selection.

After deduplication of search results, 560 abstracts were reviewed (see figure 1). In total, 468 were excluded as not eligible for our review at this stage (case reports, reviews, surgical series or description of practice, animal models, paediatric cases, pleurodesis for pleural effusions only, duplicate data or basic science articles) and we were unfortunately unable to obtain full-text copies of 13 papers published prior to 1995 (in foreign language). Of the remaining 92 potentially eligible papers, an additional 42 studies were identified as not being eligible for data extraction (20 within inadequate data or no long-term follow-up data, 6 reviews, 6 with <10 cases, 4 were duplicate data from other publications, 4 conference posters or abstracts only and 2 only related to pleural effusion management) when full texts were screened. Hence, 50 relevant studies were identified. We summarise results from 50 studies.

These 50 studies varied in size, quality and study design: 9 RCTs, 10 prospective case series (3 with non-randomised comparator groups) and 31 retrospective case series (10 with non-randomised comparator groups). The indication for pleurodesis varied; 16 (32%) studies stated an indication of pneumothorax recurrence or ongoing air leak, 7 (14%) enrolled only those at first occurrence, 6 (12%) assessed patients with ongoing air leak, 5 (10%) studied recurrent pneumothorax only and 3 studies (6%) enrolled a mixture of first occurrence, recurrent pneumothorax and ongoing leak or haemothorax. The remaining 13 studies (26%) did not specify the indication for recurrence prevention precisely enough to categorise this further.

Assessment of bias of the RCTs showed a low risk of reporting, detection or attrition bias as the outcomes of pneumothorax recurrence were well reported and the loss to follow-up was low. The randomisation process was generally well described and adequate, with the exception of some of the older studies that did not describe the process^6^ ^7^ and a more recent study that appeared to be alternating treatment arms based upon trial number.^17^ None of the studies were blinded, some providing explanation of why this was not attempted: difficulties of matching colour of agent or that the expected pain associated with minocycline instillation would be likely to unmask the blinding.

### Talc

Twenty-four studies used talc as a chemical pleurodesis agent. Twelve assessed the efficacy of talc poudrage for the treatment of PSP: four in which talc poudrage was performed at medical thoracoscopy with no associated intervention on the lung (table 1) and eight in which talc poudrage was administered post-video-assisted thoracoscopic surgery (VATS) with bleb electrocoagulation, bleb resection or apical bullectomy (table 2).

**Table 1:** Efficacy of talc pleurodesis for primary spontaneous pneumothorax at thoracoscopy (no intervention on lung)

Study author	Year	Study design	Total number of cases	Intervention (n)	Cointervention	Intervention recurrence rate (95% CI)	Control/reference arm (n)?	Control/reference recurrence rate (95% CI)	Follow-up period (months)	OR (vs control/reference) (95% CI)
*RCT*										
Tschopp^18^	2002	RCT	108	Talc poudrage (61)	Thoracoscopy only	5.1% (1.6% to 16.2%)	Drainage only (47)	34.0% (18.6% to 62.2%)	61^MN^	0.10 (0.03 to 0.38)
*Retrospective case series (with comparator group)*										
Verschoof^19^	1988	Retrospective case series	61*	Talc poudrage (38)	Thoracoscopy only	2.6% (0.4% to 19.2%)	Drainage only (23)	26.1% (10.3% to 66.2%)	48^MN^	0.08 (0.01 to 0.69)
*Retrospective case series (no comparator group)*										
Györik^20^	2007	Retrospective case series	59†	Talc poudrage (59)	Thoracoscopy‡	10.2%† (4.4% to 23.7%)	–	–	118^MD^	–
Adewole^21^	2015	Retrospective case series	21*	Talc poudrage (21)	Thoracoscopy only	9.5% (2.2% to 40.9%)	–	–	24^MN^	–

*Primary spontaneous pneumothorax patients only.

†Includes three early failures in recurrence rates.

‡Electrocoagulation performed in four cases.

MD, median; MN, mean; RCT, randomised controlled trial.

**Table 2:** Efficacy of talc pleurodesis for primary spontaneous pneumothorax: post surgery (intervention on lung)

Study author	Year	Study design	Total number of cases	Intervention (n)	Cointervention	Intervention recurrence rate (95% CI)	Control/reference arm (n)?	Control/reference recurrence rate (95% CI)	Follow-up period (months)	OR (vs control/reference) (95% CI)
*RCT*										
Chung^22^	2008	RCT (3 arms)	141	Talc and dextrose (42)	VATS+bleb resection or electrocoagulation	2.4% (0.3% to 17.3%)	Drainage only (50)	6.0% (1.9% to 19.3%)	24^MN^, 20^MN^	0.38 (0.04 to 3.82)
				Dextrose only (49)	“	2.0% (0.3% to 14.8%)			18^MN^	0.33 (0.03 to 3.25)
*Prospective series*										
Ramos-Izquierdo^23^	2010	Prospective series	133	Talc poudrage (133)	VATS±bleb electrocoagulation	3.2% (1.2% to 8.7%)	–	–	36^MN^	–
Dubois^24^	2010	Prospective series	72	Talc poudrage (72)	VATS+apical bullectomy	0% (0.0% to 4.2%)	–	–	12^MN^	–
*Retrospective case series (with comparator group)*										
Moreno-Merino^26^	2012	Retrospective case series	787	Talc poudrage (388)	VATS+bullectomy	1.5%* (0.7% to 3.5%)	Pleural abrasion† (399)	4.0%* (2.4% to 6.6%)	Unclear	0.38 (0.15 to 0.97)
Janssen^29^	1994	Retrospective case series	44	Talc poudrage (21)	VATS±bleb resection or electrocoagulation‡	0% (0.0% to 14.3%)	VATS-bullectomy (23)‡	8.7% (2.0% to 37.1%)	>18	0.20 (0.01 to 4.42)
*Retrospective case series (no comparator group)*										
Cardillo^25^	2006	Retrospective case series	861	Talc poudrage (861)	VATS±bullectomy	1.7%§ (1.0% to 3.0%)	–	–	53^MN^	–
Margolis^27^	2003	Retrospective case series	156	Talc poudrage (156)	VATS+bleb resection	0% (0.0% to 1.9%)	–	–	62^MD^	–
Mármol Cazas^28^	2011	Retrospective case series	130	Talc poudrage (130)	VATS±bullectomy	3.1% (1.1% to 8.3%)	–	–	10^MN^	–

*Includes early treatment failures requiring reintervention.

†Historical comparison.

‡Thoracoscopic talc poudrage was performed in patients with normal pleura or bullae <2 cm diameter, bullectomy was performed in those with bullae >2 cm.

§Excluded 56 lost to follow-up.

MD, median; MN, mean; RCT, randomised controlled trial; VATS, video-assisted thoracic surgery.

Of the four studies in which talc poudrage was performed without intervention on the lung (table 1), only one was an RCT. It demonstrated a lower recurrence rate among those receiving talc poudrage compared with those treated with drainage alone (talc 5.1% vs drainage 34.0%, OR 0.10, 95% CI 0.03 to 0.38).^18^ A case series study with a (non-randomised) control arm provided similar estimates: talc 2.6% versus drainage 26.1%, OR 0.08 (95% CI 0.01 to 0.69).^19^ Two case series, without comparators, estimated 10.2% recurrence in patients presenting with recurrent PSP or ongoing air leak (including early failures),^20^ and 9.5% in combination of first episode (62%) and recurrent (38%) PSPs.^21^ All four studies had follow-up periods of at least 24 months (table 1).

In the eight surgical studies evaluating talc poudrage for PSP post-VATS with bleb electrocoagulation, bleb resection or apical bullectomy, the follow-up period was variable from 10 to 62 months (table 2). There was only one RCT, which was carried out in 141 patients undergoing VATS with bleb resection or electrocoagulation. The recurrence rate among patients receiving talc and dextrose was 2.4% compared with 6.0% in the control arm of surgery only. However, the CI for the OR was very wide, indicating that we cannot be confident about this finding: OR 0.38 (95% CI 0.04 to 3.82).^22^ Further, similar results were also seen by simply introducing dextrose without talc.^22^ Observed recurrence rates across the other seven surgical studies were between 0.0% and 3.2%.^23–29^ Only two of these had (non-randomised) comparator groups,^26^ ^29^ one of which provided statistical evidence for a reduced recurrence rate in those receiving talc compared with pleural abrasion: talc 1.5% versus pleural abrasion 4.0%, OR 0.38 (95% CI 0.15 to 0.97).^26^

Indications for surgical intervention in these studies were recurrent PSP or ongoing air leak,^24^ ^25^ ^28^ including first presentation in two studies,^23^ ^29^ one solely treating first occurrence^27^ and two in which this aspect was not specified.^22^ ^26^ The surgical procedures undertaken varied both within and across the studies. Some studies stated that bleb/apical electrocoagulation or resection was only performed if visible abnormalities were seen,^23^ ^25^ ^28^ ^29^ whereas others performed the procedure in all cases.^22^ ^24^ ^26^ ^27^

The remaining 12 studies used talc to treat both patients with PSP and SSP (table 3). One RCT estimated a reduction in recurrence rates by performing talc pleurodesis via chest drain compared with drainage alone, OR 0.16 (95% CI 0.03 to 0.85).^6^ Two small studies insufflated talc under local anaesthetic and reported 0% recurrence in 24 patients^30^ and 20 patients,^31^ respectively. However, four larger series including 521 patients undergoing talc poudrage at thoracoscopy found recurrence rates of 5.6–16.1% (including early failures).^32–35^ One study comparing talc to autologous blood pleurodesis found lower recurrence in the talc group (OR 0.48) but wide CIs (0.10 to 2.24).^36^ A retrospective case series of 122 patients using talc pleurodesis via chest drain found a similar recurrence rate of 13.3%.^37^ A further three retrospective surgical studies, including 317 patients undergoing VATS bullectomy and talc poudrage for PSP and SSP, found recurrence rates of 1.1–4.5%.^38–40^ The (non-randomised) comparator groups of the two studies (n=312) found the recurrence rate for talc via chest drain for SSP to be 2.9%^38^ and 30.8%.^40^ Although the difference in the latter study was statistically significant, the two patient groups were very different: patients receiving talc via chest drain were those not deemed fit for VATS.^40^

**Table 3:** Efficacy of talc pleurodesis on spontaneous pneumothorax (PSP and SSP): medically and surgically treated

Study author	Year	Study design	Total number of cases	PSP or SSP? (n)	Intervention (n)	Intervention recurrence rate (95% CI)	Control/reference arm (n)?	Control/reference recurrence rate (95% CI)	Follow-up period (months)	OR (vs control/reference) (95% CI)
*RCTs—medical*										
Almind^6^	1989	RCT*	96*	PSP (71) SSP (25)*	Talc via chest drain (29)	8.3% (2.0% to 35.4%)	Drainage only (34)	36.0% (15.9% to 81.5%)	55^MN^	0.16 (0.03 to 0.85)
*Prospective series—medical*										
Noppen^32^	1997	Prospective case series	54	PSP (31) SSP (23)	Talc at thoracoscopy (54)±bleb electrocoagulation	7.4% (2.7% to 20.5%)	–	–	18^MN^	–
Milanez^33^	1994	Prospective case series	18	PSP (15) SSP (3)	Talc at thoracoscopy (18)	5.6% (0.7% to 41.7%)	–	–	38.5^MN^	–
*Retrospective case series—medical (with comparator group)*										
Aihara^36^	2011	Retrospective case series	36	PSP (0) SSP (36)	Talc via chest drain (14)	24.1% (6.0% to 76.8%)	Blood (22)	36.4% (15.3% to 86.7%)	15^MN^	0.48 (0.10 to 2.24)
*Retrospective case series—medical (no comparator group)*										
Van de Brekel^34^	1993	Retrospective case series	356†	Unclear	Talc at thoracoscopy (356)	12.1% (8.8% to 16.6%)	†	†	12–240	–
Weissberg^37^	1993	Retrospective case series	122	Unclear	Talc via chest drain (122)	13.3% (7.7% to 22.9%)	–	–	Unclear	–
Tschopp^35^	1997	Retrospective case series	93	PSP (65) SSP (28)	Talc at thoracoscopy (93)	16.1% (9.3% to 28.0%)	–	–	60	–
Nandi^30^	1980	Retrospective case series	24	PSP (0) SSP (24)	Talc via chest drain (24)	0% (0.0% to 12.5%)	–	–	2–24	–
Pletinckx^31^	2005	Retrospective case series	20	PSP (5) SSP (15)	Talc at thoracoscopy (54)±bleb resection	0% (0.0% to 15.0%)	–	–		–
*Retrospective case series—surgical (with comparator group)*										
Shaikhrezai^38^	1984	Retrospective case series	519	PSP (444) SSP (75)	VATS+bullectomy+talc poudrage (246)	1.2% (0.4% to 3.8%)	VATS+bullectomy+abrasion (273)	2.9% (1.5% to 5.9%)	73^MD^	0.41 (0.11 to 1.56)
Kim^40^	2011	Retrospective case series	61	PSP (0) SSP (61)	VATS+bullectomy+talc (22)	4.5% (0.6% to 33.8%)	Drainage+talc via drain (39)	30.8% (15.6% to 60.7%)	Unclear	0.11 (0.01 to 0.89)
*Retrospective case series—surgical (no comparator group)*										
de Campos^39^	2001	Retrospective case series	49	Unclear	VATS+bullectomy+talc (49)	2.0% (0.3% to 14.8%)	–	–	24–60	–

Follow-up range shown if no average given.

*RCT had three arms (total numbers include tetracycline arm, n=33).

†Thoracotomy and bullectomy were performed in those with bullae >2 cm at thoracoscopy—not included in analysis.

MD, median; MN, mean; PSP, primary spontaneous pneumothorax; RCT, randomised controlled trial; SSP, secondary spontaneous pneumothorax; VATS, video-assisted thoracic surgery.

### Tetracycline

Eleven studies evaluated the efficacy of tetracycline pleurodesis for SP via chest drains or thoracoscopy without intervention on the lung (table 4). These were of variable quality including both PSP and SSP but comprised three RCTs including a total of 366 patients. Two RCTs, from 1990 and 1989, randomised patients to either tetracycline versus chest drain or drainage only.^6^ ^7^ Both reported lower rates in the tetracycline arm but only one study was statistically significant, OR 0.48 (95% CI 0.27 to 0.85).^7^ The other small RCT of tetracycline versus silver nitrate at thoracoscopy found recurrence rate of 0% in both arms.^41^ Four non-randomised retrospective studies of tetracycline versus drainage alone all estimated reduced recurrence rates in the tetracycline group. However, all but one had wide CIs crossing the null value of 1: OR 0.50 (95% CI 0.23 to 1.09), 0.25 (95% CI 0.09 to 0.73), 0.43 (95% CI 0.07 to 2.68) and 0.14 (95% CI 0.01 to 2.53).^42–45^ It should be noted that four studies included only patients with first occurrence of SP,^6^ ^41^ ^43^ ^45^ with the remaining seven studies not specifying indication for recurrence prevention. One surgical study of tetracycline after VATS bullectomy reported no recurrences (0.0%) in contrast to 10.9% in a group treated non-surgically with chest tube drainage alone.^46^ However, another older prospective surgical series showed a recurrence rate of 9.4% with no control group.^47^

**Table 4:** Efficacy of tetracycline pleurodesis for spontaneous pneumothorax (no intervention on lung)

Study author	Year	Study design	Total number of cases	PSP or SSP? (n)	Intervention (n)	Intervention recurrence rate (95% CI)	Control/reference arm (n)?	Control/reference recurrence rate (95% CI)	Follow-up period (months)	OR (vs control/reference) (95% CI)
*RCTs—medical*										
Light^7^	1990	RCT	229	PSP (46) SSP (183)	Tetracycline via chest drain (113)	25.0% (16.2% to 38.0%)	Drainage only (116)	40.7% (28.0% to 58.7%)	29–34^MN^	0.48 (0.27 to 0.85)
Almind^6^	1989	RCT*	96*	PSP (71) SSP (25)*	Tetracycline via chest drain (33)	13.0% (3.9% to 43.9%)	Drainage only (34)	36.0% (15.9% to 81.5%)	55^MN^	0.27 (0.06 to 1.15)
Wied^41^	1983	RCT	41	PSP (41)	Tetracycline at thoracoscopy (18)	0% (0.0% to 16.7%)	Silver nitrate (22)	0% (0.0% to 13.6%)	14^MD^	–
*Prospective series—medical*										
Alfageme^42^	1994	Prospective case series	146	PSP (96) SSP (50)	Tetracycline via chest drain (78)	18.9%† (10.6% to 33.8%)	Drainage only (68)	35.3% (19.9% to 62.7%)	45^MN^	0.43 (0.19 to 0.97)
*Prospective series—surgical*										
Waterworth^47^	1995	Prospective series	32	PSP (32)	VATS+bullectomy+tetracycline (32)	9.4% (2.9% to 30.8%)	–	–	19^MD^	–
*Retrospective case series—medical (with comparator group)*										
Guo^43^	2005	Retrospective case series	138	PSP (86) SSP (52)	Tetracycline via chest drain (45)	33.3% (17.9% to 62.0%)	Drainage only (70)	50.0% (31.3% to 79.9%)	6–69	0.50 (0.23 to 1.09)
Tanaka^44^	1993	Retrospective case series	78‡	SSP (78)	Tetracycline via chest drain (32)	18.8% (7.7% to 45.6%)	Drainage only (46)	47.8% (26.8% to 85.3%)	48^MN^	0.25 (0.09 to 0.73)
van den Brande^45^	1989	Retrospective case series	20	PSP (20)	Tetracycline and 30% glucose via chest drain (10)	30.0% (7.8% to 100%)	Drainage only (10)	50.0% (14.5% to 100%)	26^MN^ (intervention) and 18^MN^ (control)	0.43 (0.07 to 2.68)
*Retrospective case series—surgical (with comparator group)*										
Lee^46^	2008	Retrospective case series	91	PSP (91)	VATS+bullectomy+tetracycline (27)	0.0% (0.0% to 11.1%)	Drainage only (64)	10.9% (5.0% to 24.0%)	16^MN^	0.14 (0.01 to 2.53)
*Retrospective case series—medical (no comparator group)*										
Olsen^64^	1992	Retrospective case series	390§	PSP (390)	Tetracycline at thoracoscopy (390)	15.6% (11.9% to 20.6%)	§	§	43^MD^	–
Primrose^65^	1984	Retrospective case series	19¶	Unclear	Tetracycline via chest drain (19)	47.4% (19.2% to 100%)	–	–	Unclear	–

Follow-up range shown if no average given.

*RCT had three arms (total numbers include talc arm, n=29).

†Includes eight early treatment failures.

‡Excluding patients being observed or aspirated only and those having thoracotomy.

§Thoracotomy and bullectomy were performed in those with bullae >2 cm at thoracoscopy—not included in analysis.

¶Small subgroup of patients undergoing pleurodesis.

MD, median; MN, mean; PSP, primary spontaneous pneumothorax; RCT, randomised controlled trial; SSP, secondary spontaneous pneumothorax; VATS, video-assisted thoracic surgery.

### Blood

Five studies of blood patch pleurodesis were included in the analysis (n=270, table 5). There were no RCTs compared with drainage alone. Two studies prospectively enrolled patients after SP with persistent air leak. One non-randomised study estimated an OR of 0.47 (95% CI 0.17 to 1.32) for recurrence in patients following autologous blood pleurodesis compared with drainage alone (15.6% blood pleurodesis, including early failures requiring surgery, vs 28.1% drainage).^48^

**Table 5:** Efficacy of blood pleurodesis for spontaneous pneumothorax (PSP and SSP) via chest drain

Study author	Year	Study design	Total number of cases	PSP or SSP? (n)	Intervention (n)	Intervention recurrence rate (95% CI)	Control/reference arm (n)?	Control/reference recurrence rate (95% CI)	Follow-up period (months)	OR (vs control/reference) (95% CI)
*Prospective series*										
Cagirici^48^	1998	Prospective series	167	PSP (116) SSP (51)	Blood via chest drain (32)	15.6% (6.0% to 40.6%)	Drainage only (135)	28.1% (19.3% to 41.0%)	12–48	0.47 (0.17 to 1.32)
Ando^66^	1999	Prospective series	11	SSP (11)	Blood via chest drain (11)	18.2% (3.9% to 84.1%)	–	–	2–24	–
*Retrospective series*										
Aihara^36^	2011	Retrospective case series	36	SSP (36)	Blood via chest drain (22)	36.4% (15.3% to 86.7%)	Talc (14)	21.4% (6.0% to 76.8%)	15^MN^	2.10 (0.45 to 9.81)
Evman^50^	2016	Retrospective case series	31	SSP (31)	Blood via chest drain (31)	16.1%* (6.2% to 42.0%)	–	–	29^MN^	–
Robinson^49^	1987	Retrospective case series	25	Unclear	Blood via chest drain (25)	16.0% (5.5% to 46.6%)	–	–	24–132	–

Follow-up range shown if no average given.

*Includes early treatment failures.

MN, mean; PSP, primary spontaneous pneumothorax; SSP, secondary spontaneous pneumothorax.

As noted in the ‘Talc’ section above, a small retrospective comparison study of talc via chest drain versus blood patch was inconclusive.^39^ Two further small retrospective series of patients unfit for surgery with recurrence or persistent air leak found long-term recurrence of 16.0%^49^ and 16.1%^50^ with no comparator groups.

### Minocycline

Three RCTs in patients with PSP (n=498) found recurrence rates of 0%^17^ and 1.9% after the instillation of minocycline after lung re-expansion post-VATS procedure^51^ but 29.2% after instillation via chest drain only (ie, medical management, no intervention on the lung) in patients with first presentations of PSP^52^ (table 6). This RCT provided evidence of a reduction in recurrence compared with drainage only in non-surgical patients (minocycline 29.2% vs drainage only 49.1%, OR 0.43, 95% CI 0.24 to 0.75).^52^ Among surgical patients, Chen *et al*^51^ also observed a reduced recurrence rate compared with patients receiving saline, but the strength of statistical evidence was weak (minocycline 1.9% vs saline 8.1%, OR 0.23, 95% CI 0.05 to 1.09). The third surgical RCT, comparing minocycline versus mechanical abrasion, was small and inconclusive (minocycline 0.0% vs mechanical abrasion 5.0%, OR 0.18, 95% CI 0.01 to 3.89).^17^

**Table 6:** Efficacy of minocycline pleurodesis for primary spontaneous pneumothorax: medical and surgical

Study author	Year	Study design	Total number of cases	Intervention (n)	Cointervention	Intervention recurrence rate (95% CI)	Control/reference arm (n)?	Control/reference recurrence rate (95% CI)	Follow-up period (months)	OR (vs control/reference) (95% CI)
*RCT—medical*										
Chen^52^	2013	RCT	214	Minocycline via chest drain (106)	Nil	29.2% (19.2% to 44.4%)	Drainage only (108)	49.1% (33.7% to 71.6%)	19^MN^	0.43 (0.24 to 0.75)
*RCT—surgical*										
Chen^51^	2006	RCT	202	Minocycline via chest drain (103)*	VATS+bullectomy	1.9% (0.5% to 7.9%)	No agent (99)	8.1% (3.9% to 16.6%)	29^MN^	0.23 (0.05 to 1.09)
Alayouty^17^	2011	RCT	82	Minocycline via chest drain (42)*	VATS+bullectomy	0% (0.0% to 7.1%)	Mechanical abrasion (40)	5.0% (1.2% to 20.7%)	36^MN^	0.18 (0.01 to 3.89)
*Retrospective case series—surgical*										
Chen^53^	2004	Retrospective case series	364	Minocycline via chest drain (313)*	VATS+bullectomy	2.9% (1.5% to 5.6%)	Saline (51)†	9.8% (3.9% to 24.7%)	48^MN^	0.27 (0.09 to 0.85)
How^54^	2014	Retrospective case series	79‡	Minocycline via chest drain (60)	VATS+bullectomy	36.7% (21.7% to 62.0%)	OK-432 (19)	5.3% (0.7% to 39.4%)	16^MN^	10.42 (1.30 to 83.50)

*Minocycline introduced postsurgery once lung had re-expanded.

†Historical comparison.

‡Only patients with postoperative air leak after VATS were included.

RCT, randomised controlled trial; VATS, video-assisted thoracic surgery.

There were also two retrospective non-randomised studies with control arms. One suggested that minocycline significantly reduced recurrence rates with minocycline post-VATS compared with saline (minocycline 2.9% vs 9.8% saline, OR 0.27, 95% CI 0.09 to 0.85) in a historical comparison.^53^ The other comparative study in patients with prolonged air leak post-VATS for SP found increased recurrence rates in the minocycline group compared with OK-432 (estimated failure rate 36.7% minocycline vs 5.3% OK-432, OR 10.42, 95% CI 1.30 to 83.5).^54^

### Other agents

A retrospective surgical series of 81 cases reported a recurrence rate of 6.2% when using iodopovidone during VATS despite only 37% undergoing bullae resection. Two small studies in India assessed the efficacy of iodopovidone via chest drain. One randomised trial (n=35) had no recurrence in either the iodopovidone or talc pleurodesis arms^55^ and a retrospective review of 27 cases found a recurrence rate of 7.4%.^56^

Two studies used silver nitrate as a chemical pleurodesis agent: one RCT (see table 4) reported 0% recurrence after instillation at thoracoscopy but described increased pleural fluid production and longer hospital stay than using tetracycline.^41^ A retrospective surgical study (n=184, 3 years follow-up) used silver nitrate after VATS bullectomy with a recurrence rate of 1.1%, but had no comparison group.^57^ A retrospective series reviewing recurrence prevention at first occurrence found a significant recurrence rate using gentamicin via chest drain, although this was more efficacious than drainage alone (3-year recurrence rates 26.1% and 50.0%, respectively (OR 0.35, 95% CI 0.12 to 1.00, with a reported p value <0.05).^43^ A small study including 17 spontaneous pneumothoraces found only one recurrence (5.9%) after administration of quinacrine.^58^ In a group of 57 patients with SSP deemed too high risk for surgery with ongoing air leak, fibrin glue (diluted fourfold) was instilled via chest drain. The long-term (60 months) recurrence rate was 10.5%.^59^ The addition of acromycin post-VATS bullectomy reportedly reduced recurrence to 3.8% from 20.0%, in a non-randomised historical comparison group, but CIs were wide (OR 0.16, 95% CI 0.02 to 1.48), despite the authors stating a p value <0.05.^60^

## Discussion

This is the first study to our knowledge to systematically review the evidence for the effectiveness of pleurodesis in recurrence prevention for all chemical pleurodesis agents in cases of SP in both ‘medical’ pleurodesis (ie, no intervention on the lung) and as an adjunct to surgical procedures. Patients with postoperative surgical air leak were specifically excluded as patients having undergone thoracic surgery (eg, for wedge resection or lobectomy) with subsequent air leak are likely to be a different population from those with spontaneous pneumothoraces. For the same reason, unlike previous reviews of pleurodesis efficacy, patients undergoing pleurodesis for malignant pleural effusion recurrence prevention were excluded.

Given the considerable heterogeneity across studies in design, outcomes and interventions, formal data synthesis via meta-analysis was not conducted as we do not believe the results would be clinically meaningful. Only 9 of 50 studies were RCTs. Also, 13 of the 41 other studies (case series) provided comparator groups but these were historical comparisons or non-randomised comparator groups. The lack of head-to-head comparisons limits the ability to formally compare relative effectiveness of different agents in this review. This is in contrast to a recent Cochrane review of pleurodesis agents in malignant pleural effusion, in which a network meta-analysis of pleurodesis agents was possible with 62 RCTs identified.^61^

Studies in which the control arms were drainage with a chest drain only (ie, no other agent) suggest recurrence rates of 26.1–50.1%. Talc pleurodesis would appear to be effective at reducing recurrence for PSP when used via poudrage at thoracoscopy, with two studies with comparator arms giving recurrence rates of 5.1% and 2.6% with OR 0.10 (95% CI 0.03 to 0.38) and 0.08 (95% CI 0.01 to 0.69), respectively, when comparing talc poudrage to drainage alone.^18^ ^19^ However, more recent case series data suggested higher recurrence rates (9.5%^20^ and 10.2%).^21^

When talc is used in patients undergoing surgical (VATS) procedures, the recurrence rate appears to be low (between 0.0% and 3.2%) and it seems that the addition of talc contributes to a lower recurrence compared with VATS bullectomy and either drainage or abrasion alone. There have been no direct comparisons of VATS bullectomy and pleurectomy to VATS bullectomy and talc pleurodesis. Results for talc poudrage at thoracoscopy (without interventional on the lung) in the general SP (PSP and SSP) appear to be between 5.6% and 16.1% in larger case series (without comparator arms). No studies of talc slurry pleurodesis for SP alone were found, except two studies with small comparator groups of 14 and 10 patients, which gave 21.4% and 0.0% recurrence rates, respectively.^36^ ^55^ A previous systematic review of talc pleurodesis of 22 studies in 1994 found an overall success rate of 91%; however, 6 of 15 studies assessing talc poudrage and the 4 studies assessing talc slurry were small (≤10 patients) such that estimated rates are very imprecise.^62^

The majority of studies assessing the reduction in recurrence rate by ‘medical’ pleurodesis agents via chest drain involve tetracycline. The higher-quality studies (RCTs) would suggest a recurrent rate of between 13% and 25%, which is significantly better than those receiving drainage only (OR 0.27, 95% CI 0.06 to 1.15, and 0.61 with 0.48, 95% CI 0.27 to 0.85).^6^ ^7^ Blood patch pleurodesis for persistent air leak in those patients not fit for surgery seems to deliver a recurrence rate of around 16%.^49^ ^50^ An RCT assessing the short-term efficacy of autologous blood patch pleurodesis at varying doses found that administration of 1 or 2 mL/kg was more successful at ceasing air leak by 13 days (both 82%) than 0.5 mL/kg or saline (27% and 9%, respectively).^63^

Minocycline appears to be the agent of choice in Taiwan. Instillation of talc via chest drain once the lung has re-expanded post-VATS bullectomy results in a low recurrence rate.^17^ ^51^ ^53^ Minocycline via chest drain without surgical intervention in patients with first presentation of PSP also seems to provide a significant reduction in recurrence,^52^ although its use is not commonplace.

There are numerous other pleurodesis agents with potential efficacy. Iodopovidone is widely available in India with one small RCT demonstrating equivalent success rates to talc.^55^ The case for widespread use of acromycin, gentamycin or quinacrine via chest drains, and silver nitrate or diluted fibrin glue post-VATS is still to be made.

These results are consistent with recent network meta-analysis of pleurodesis for malignant pleural effusions, which found that talc poudrage was highly effective, followed by talc slurry, mepacrine, iodine, bleomycin and doxycycline, although this is clearly a different patient population.^61^

The indication for pleurodesis in the vast majority of studies was ongoing air leak or recurrence prevention at second occurrence as per guideline recommendation. However, one study assessed early surgical intervention at first occurrence.^27^ This group was controversially managed without aspiration or chest tube drainage, undergoing VATS with bleb resection and talc poudrage pleurodesis within 12 hours of first presentation. While none of these patients had a subsequent recurrence, it is likely that a significant proportion were unnecessarily operated upon as many may have resolved with conservative management and not recurred.

There are a number of limitations to this systematic review. First, our review is limited by the quality of the available data. Although we identified a few well-conducted RCTs, the majority of identified studies were observational, many of which were non-comparative. Many included studies were also retrospective, with a high risk of reporting bias. Thirteen papers published pre-1995 in foreign (non-English) language and not available in print were not included. Indications for pleurodesis varied across the studies including those solely assessing patients at first occurrence, those with recurrent pneumothorax or air leak patients, and those with ongoing air leak only. Size of pneumothorax at presentation and details of previous treatment were not always provided. There was variation both within and across surgical studies as to the specific co-incident procedure being undertaken (alongside chemical pleurodesis). The exact procedure was often determined on visual inspection of the lung (eg, bleb/bullae resection or electrocoagulation only performed if visible blebs and bullae seen) but results usually only reported as overall recurrence rates. This results in significant clinical heterogeneity in the published data, and therefore interpretation of results across study types should be guarded.

## Conclusion

This comprehensive systematic review of the literature demonstrates that numerous agents have been used for chemical pleurodesis for recurrence prevention in SP. Chemical pleurodesis alongside surgical treatment or via thoracoscopy appears most effective in preventing recurrence, but is not suitable for all patients. Evidence for relative success rates between agents is limited by the small number of randomised and prospective comparative trials. Well-controlled and conducted RCTs are now required using a number of candidate agents to assess optimal management in SP recurrence prevention and treatment.
